# Supplementary material for: Complex‐centric proteome profiling by SEC‐SWATH‐MS
Source: Mol Syst Biol. 2019 Jan 14;15(1):e8438. doi: 10.15252/msb.20188438 (PMC6346213; doi:10.15252/msb.20188438)
Supplement: Supplementary file 8 — Dataset EV7 [file MSB-15-e8438-s008.zip › feature_plots_string/Q9UBC3.pdf]

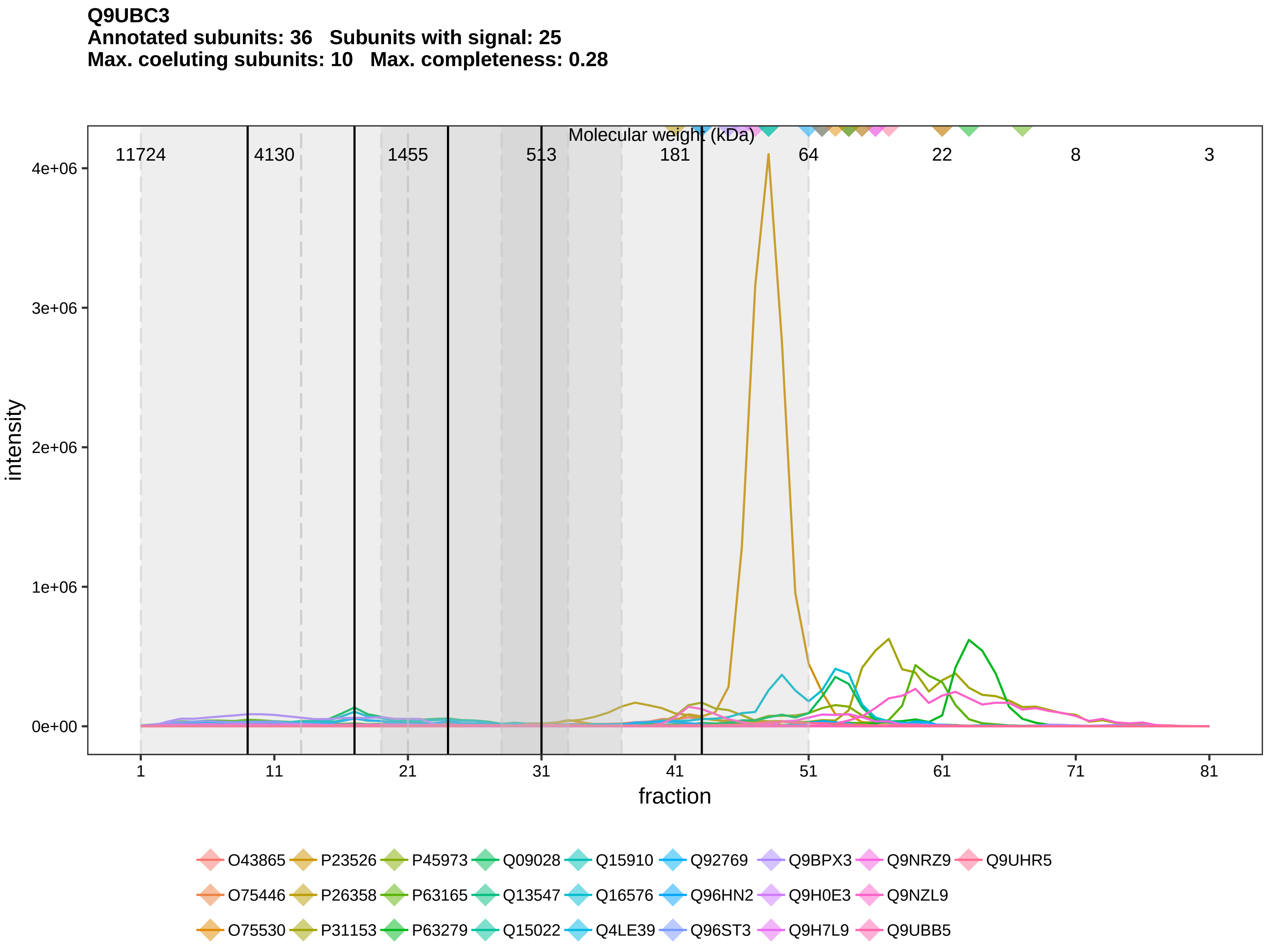

Q9UBC3
Annotated subunits: 36 Subunits with signal: 25
Max. coeluting subunits: 10 Max. completeness: 0.28
Molecular weight (kDa)
11724
4130
1455
513
181
64
22
8
3
intensity
4e+06
3e+06
2e+06
1e+06
0e+00
fraction
1
11
21
31
41
51
61
71
81
O43865
P23526
P45973
Q09028
Q15910
Q92769
Q9BPX3
Q9NRZ9
Q9UHR5
O75446
P26358
P63165
Q13547
Q16576
Q96HN2
Q9H0E3
Q9NZL9
O75530
P31153
P63279
Q15022
Q4LE39
Q96ST3
Q9H7L9
Q9UBB5
